# Humic-acid-driven escape from eye parasites revealed by RNA-seq and target-specific metabarcoding

**DOI:** 10.1186/s13071-020-04306-9

**Published:** 2020-08-28

**Authors:** Kristina Noreikiene, Mikhail Ozerov, Freed Ahmad, Toomas Kõiv, Siim Kahar, Riho Gross, Margot Sepp, Antonia Pellizzone, Eero J. Vesterinen, Veljo Kisand, Anti Vasemägi

**Affiliations:** 1 Chair of Aquaculture, Institute of Veterinary Medicine and Animal Sciences, Estonian University of Life Sciences, Kreutzwaldi 46, 51006 Tartu, Estonia; 2 Department of Biology, University of Turku, 20014 Turku, Finland; 3 Department of Aquatic Resources, Institute of Freshwater Research, Swedish University of Agricultural Sciences, 17893 Drottningholm, Sweden; 4 Biodiversity Unit, University of Turku, 20014 Turku, Finland; 5 Chair of Hydrobiology and Fishery, Institute of Agricultural and Environmental Sciences, Estonian University of Life Sciences, Kreutzwaldi 5, 51006 Tartu, Estonia; 6 Department of Life Sciences and Biotechnology, University of Ferrara, 44121 Ferrara, Italy; 7 Department of Ecology, Swedish University of Agricultural Sciences, 75651 Uppsala, Sweden; 8 Institute of Technology, University of Tartu, Nooruse 1, 50411 Tartu, Estonia

**Keywords:** Diplostomidae, Host-parasite interaction, Humic substances, Metabarcoding, *Perca fluviatilis*, RNA-seq

## Abstract

**Background:**

Next generation sequencing (NGS) technologies are extensively used to dissect the molecular mechanisms of host-parasite interactions in human pathogens. However, ecological studies have yet to fully exploit the power of NGS as a rich source for formulating and testing new hypotheses.

**Methods:**

We studied Eurasian perch (*Perca fluviatilis*) and its eye parasite (Trematoda, Diplostomidae) communities in 14 lakes that differed in humic content in order to explore host-parasite-environment interactions. We hypothesised that high humic content along with low pH would decrease the abundance of the intermediate hosts (gastropods), thus limiting the occurrence of diplostomid parasites in humic lakes. This hypothesis was initially invoked by whole eye RNA-seq data analysis and subsequently tested using PCR-based detection and a novel targeted metabarcoding approach.

**Results:**

Whole eye transcriptome results revealed overexpression of immune-related genes and the presence of eye parasite sequences in RNA-seq data obtained from perch living in clear-water lakes. Both PCR-based and targeted-metabarcoding approach showed that perch from humic lakes were completely free from diplostomid parasites, while the prevalence of eye flukes in clear-water lakes that contain low amounts of humic substances was close to 100%, with the majority of NGS reads assigned to *Tylodelphys clavata.*

**Conclusions:**

High intraspecific diversity of *T. clavata* indicates that massively parallel sequencing of naturally pooled samples represents an efficient and powerful strategy for shedding light on cryptic diversity of eye parasites. Our results demonstrate that perch populations in clear-water lakes experience contrasting eye parasite pressure compared to those from humic lakes, which is reflected by prevalent differences in the expression of immune-related genes in the eye. This study highlights the utility of NGS to discover novel host-parasite-environment interactions and provide unprecedented power to characterize the molecular diversity of cryptic parasites.
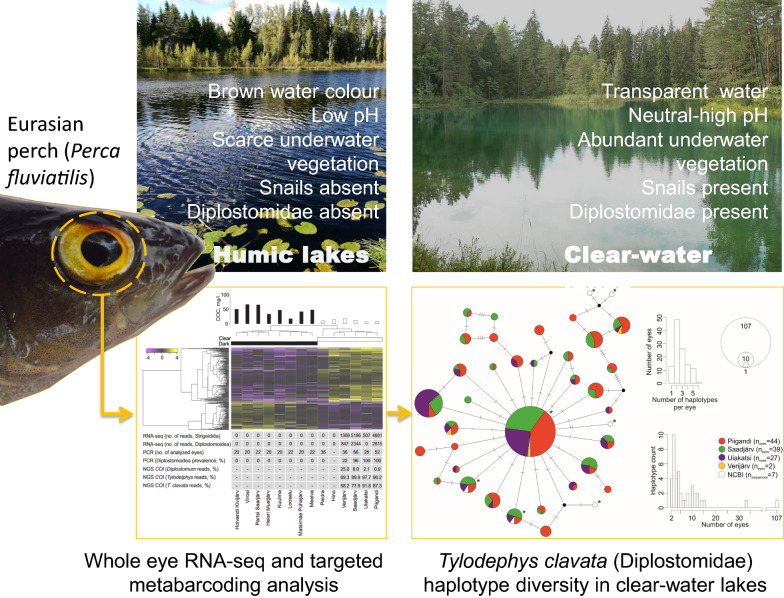

## Background

The evolutionary arms-race between host and parasites is of a key importance for maintaining species diversity and community composition. However, the pace of evolutionary change in host-parasite systems is modulated not only by co-interacting communities, but also by common components of their extrinsic environment [1–3]. Yet, the role of environment in shaping host-parasite interactions is poorly understood [4]. The advancement of next-generation sequencing (NGS) technologies provides opportunities to expand our understanding about such complex interactions at an unprecedented speed [5, 6].

During the last decade, high-throughput RNA sequencing (RNA-seq) has been increasingly used to explore infection, disease- and stress-related changes in gene expression of the host. Gene expression analyses at the whole transcriptome level have also shed light on fundamental aspects of host and parasite biology [e.g. 7, 8] and host-parasite interactions [9–12]. In addition, novel insights into the dynamics of host-parasite interactions at the molecular level are increasingly gained also by analysing sequence data that were traditionally deemed to be invaluable and hence excluded [13]. For example, a typical bioinformatics analysis pipeline involves a step where reads from DNA or RNA sequencing are aligned to the target species genome; those that do not align are simply discarded. This principle is integrated into the majority of existing pipelines because unmapped reads could originate from library contamination and sequencing errors. As such, much effort has been put towards sorting out this type of nuisance information [13, 14]. However, there is growing awareness that some of the unmapped reads could actually harbour novel genetic and ecological information. Thus far, unmapped reads from RNA- or DNA-seq data have been used to discover symbionts, pathogens, and undescribed features of the target species genome, such as highly divergent regions or insertions of the reference genome that would have been missed otherwise [13, 15–18]. Given that parasite and pathogen RNA typically represent only a tiny proportion of the total RNA of the host, very deep sequencing is necessary to obtain comprehensive understanding of the pathogen transcriptomes and genetic diversity. This means that by using untargeted sequencing of the host transcriptome it is rarely possible to obtain enough power for pathogen community composition analyses. As an alternative, a targeted amplicon-based high-throughput sequencing, known as metabarcoding, has become an essential tool for monitoring biodiversity [19, 20] and also increasingly used for understanding parasite diversity in host tissues and environmental samples [e.g. 21, 22]. Community metabarcoding is a sensitive technique that allows detection of rare and cryptic species and species associations [23, 24] as well as analyses of within species genetic variability and population structuring [25].

Diplostomidae is a geographically widely distributed trematode parasite group of species with complex life-cycles which include two intermediate hosts, lymnaeid snails and fishes, while a piscivorous bird usually serves as a definitive host. After infecting and completing its development in the snail, cercariae enter fish through the gills and skin, before settling in fish-eye structures and sometimes neural tissues. This may lead to changes in host behaviour, such as reduced feeding efficiency which decreases the body condition of the fish ([26] but see [27]). Species of the Diplostomidae are morphologically extremely difficult to distinguish and each fish may be infected by hundreds of parasites. As a result, estimating species diversity, community composition, host-parasite interaction and effects of environmental factors in this group is challenging [28–30]. While the use of molecular approaches and especially *cox*1 fragment-based species identification *via* Sanger sequencing [31] have advanced the field by revealing hidden species diversity, most of the studies have focused on describing species from single fluke isolates [30, 32, 33]. However, using single fluke sequencing is suboptimal for characterizing community composition and intraspecific genetic diversity. Massive parallel sequencing with whole tissue extracts from the host represents a potentially powerful strategy to improve characterization of both inter- and intraspecific diversity of parasites [29].

Here, we describe how initial transcriptome screening of fish eyes, where we used both host-specific and unmapped RNA-seq reads, invoked a novel hypothesis that humic-associated differences among lakes affect the prevalence of diplostomid eye parasites in the Eurasian perch (*Perca fluviatilis*). In particular, by building on RNA-seq read data and expanding upon previous work on eye parasites in perch [34], we hypothesized that the elevated content of humic substances (often measured as dissolved organic carbon (DOC) concentrations and spectral parameters of the water) would have a negative effect on the abundance of the intermediate hosts of eye flukes, gastropods, primarily *via* combined effects of low pH and water transparency affecting underwater plant growth [35]. We tested the potential link between humic substances and occurrence of diplostomid eye parasites by conducting extensive molecular screening of eye flukes and developing a targeted metabarcoding approach to efficiently screen intra- and interspecific genetic diversity of parasites from host eye tissue.

## Methods

### Sample collection

Perch sampling was carried out in 8 humic and 6 clear-water lakes in Estonia in 2016 and 2017 (Additional file 1: Figure S1, Additional file 2: Table S1). Fish were sacrificed by an overdose of tricaine methanesulfonate (MS-222), individually labelled, and their eyes were enucleated and snap frozen in liquid nitrogen. Surface water samples from each lake were collected during the sampling in 2016 and pH, DOC concentrations to characterize the humic content of lakes, and different spectral parameters were determined (Additional file 2: Table S6) as described previously [36]. The diversity of gastropods in 10 out of the 14 studied lakes was obtained from the Estonian Environmental Monitoring database (Table 1, https://www.keskkonnaagentuur.ee). The details on sampling protocol and subsequent NGS analyses are provided in the Additional file 3: Text S1.

**Table 1:** Study lake characteristics and gastropod occurrence data

Lake	Type	Geographical coordinates	Water chemistry				Gastropod species occurence^a^		
			Dissolved organic carbon (DOC, mg/l)	Freshness index	Fluorescence index	pH	No. of sampling visits	Sampling year	Gastropod species
Holvandi Kivijärv	H	58.0410°N, 27.1965°E	50.04	0.38	1.27	6.30	1	2012	None
Virosi	H	58.0259°N, 27.2551°E	66.1	0.34	1.26	5.45	7	1995–2012	None
Partsi Saarjärv	H	57.9978°N, 27.1662°E	64.8	0.34	1.25	5.30	6	1995–2012	None
Heisri Mustjärv	H	58.0249°N, 26.8312°E	33.28	0.43	1.29	8.20	na	na	na
Kuulma	H	57.9569°N, 27.1613°E	47.1	0.41	1.30	4.50	na	na	na
Loosalu	H	58.9361°N, 25.0824°E	17.41	0.44	1.28	4.70	14	Multiple	None
Matsimäe Pühajärv	H	59.0611°N, 25.5135°E	41.63	0.39	1.22	na	na	na	na
Meelva	H	58.1407°N, 27.3852°E	47.77	0.38	1.28	5.60	7	1994-1995	*Anisus vortex*, *Planorbis corneus*
Paidra	CW	57.9110°N, 27.1910°E	10.23	0.68	1.39	6.70	na	na	na
Hino	CW	57.5766°N, 27.2298°E	13.91	0.84	1.56	8.65	2	2001	*Lymnaea stagnalis*
Verijärv	CW	57.8106°N, 27.0470°E	16.78	0.73	1.53	8.50	1	2002	*Lymnaea stagnalis*, *Ancylus fluviatilis*, *Valvata piscinalis*, *Bithynia tentaculata*
Saadjärv	CW	58.5535°N, 26.6059°E	11.24	0.75	1.51	8.75	14	Multiple	*Lymnaea stagnalis*, *Bithynia tentaculata*, *Physa fontinalis*, *Radix balthica*, *Myxas glutinosa*, *Valvata piscinalis*, *Valvata pulchella*, *Valvata depressa*, *Gyraulus albus*, *Anisus vortex*
Uiakatsi	CW	57.9532°N, 26.6365°E	6.684	0.72	1.46	8.35	2	2007–2012	*Lymnaea stagnalis*, *Hippeutis complanatus*
Piigandi	CW	58.0176°N, 26.7913°E	8.337	0.73	1.48	7.00	1	2012	None

^a^Snail occurrence data was obtained from Estonian Environmental Monitoring database (https://www.keskkonnaagentuur.ee)

*Abbreviations*: H, humic-water lake; CW, clear-water lake; none, no gastropod species observed; na, data not available

### RNA expression and unmapped read analysis

Total RNA was extracted from the whole eye tissues collected in 2016, and libraries were sequenced with Illumina HiSeq 3000 (Illumina Inc., San Diego, USA). Reads that passed quality control were mapped onto the reference genome of *Perca fluviatilis* [37] using hisat 2 2.1.0 [38] (Additional file 3: Text S1). Differential expression analysis between the two groups of lakes (humic *vs* clear-water) was performed using the *DEseq2* package 1.22.2 [39] in R 3.3.4 [40]. All genes with an adjusted *P*-value ≤ 0.05 [41] were considered as significantly differentially expressed between populations from the two groups of lakes. Human orthologue gene symbols were searched for using complete gene names in NCBI. Gene Ontology (GO)-enrichment analysis of differentially expressed genes against all orthologous gene symbols as a background was performed using Gorilla [42]. The GO terms with a false discovery rate (FDR) ≤ 0.05 were considered as significant.

Unmapped reads from each sample were further analysed to detect the occurrence of parasite reads among the whole-eye RNA-seq data. Briefly, NCBI’s blastn 2.6.0 [43] was applied to align the non-redundant sets of unmapped reads to the sequences in a non-redundant nucleotide database. To reveal the presence of the eye fluke parasites’ sequences (Trematoda: Digenea: Diplostomidae) among the unmapped reads, the taxonomic analysis of blastn outputs was processed in Megan Community Edition 6.8.18 [44].

### PCR-based confirmation of diplostomids in perch eye

DNA was extracted from the whole eye using a standard salt extraction method [45], and PCR-based screening was performed in 212 perch eye samples (Additional file 2: Table S1) using diplostomid-specific primers that amplified a fragment of the cytochrome *c* oxidase subunit 1 (*cox*1) gene [31]. Primers were modified to include linkers for Illumina-compatible adapters at their 5’-ends [46, 47]. Both eyes were screened in 172 individuals, while only the left eye was screened in the remaining 40 individuals. PCR products were visualised on a 1.5% agarose gel, and the presence of a ~500-bp amplification product was recorded as evidence of Diplostomidae infection in a given eye (Additional file 2: Table S1).

### Metabarcoding of the diplostomid community in perch eye

We used whole eyes as the starting material for the analysis; that is, the diplostomids were not individually extracted from the eye, but rather sequenced together as a naturally pooled sample [29]. Libraries were prepared from SPRI-bead-purified PCR products of 142 diplostomid-positive samples identified with the PCR described above (Additional file 2: Table S1) by attaching Illumina adapters and unique individual indices following the PCR protocol described in [47] with minor modifications (see Additional file 3: Text S1). Samples were pooled and sequenced using an Illumina MiSeq instrument (Illumina Inc., San Diego, California, USA) at the Turku Centre for Biotechnology (Turku, Finland). The paired-end raw reads were demultiplexed (Additional file 2: Table S2) and merged using PEAR 0.9.6 [48]. For robust downstream analysis, we followed a conservative approach, i.e. only the samples containing ≥ 1000 sequences [49] were retained (115 of 142; Additional file 2: Table S2).

Taxonomic classification was performed with Kraken 2.0.6-beta [50]. In addition, to validate the Kaken results with a probabilistic approach the sequences were classified by applying a naïve Bayesian classifier using RPD 11.5 [51] following [52]. For both classifiers, a custom database was generated using the available *cox*1 gene sequences for Platyhelminthes from NCBI GenBank (https://www.ncbi.nlm.nih.gov/; see details in Additional file 3: Text S1). As both taxonomic classifiers showed consistent results, the further analyses are based only on the Kraken classification. To avoid biases related to unequal number of reads per sample [53], the presence of a particular parasite genus/species in a sample was considered as highly supported if ≥ 5% of the sequences were assigned to that parasite genus/species per eye sample.

**Fig. 1:**
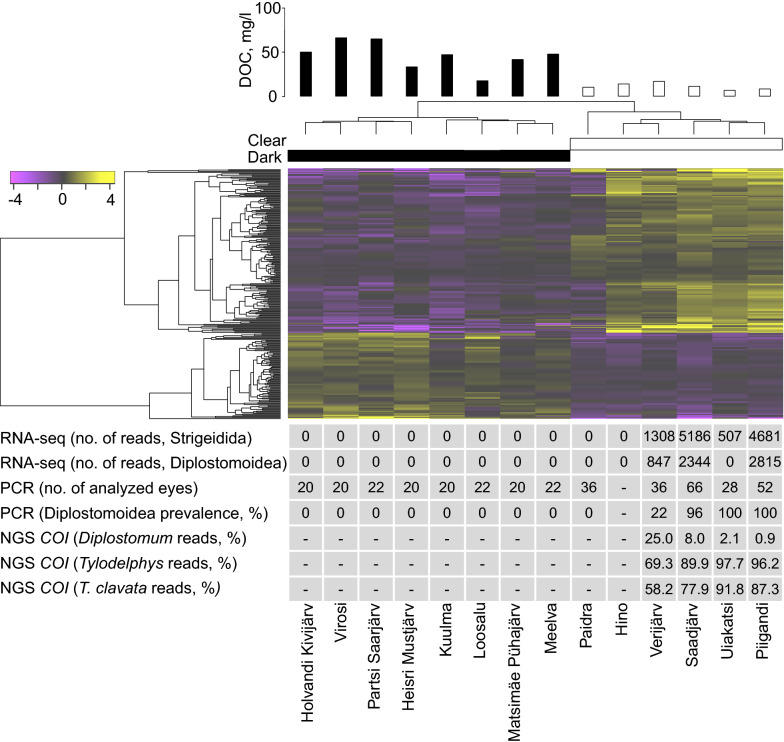
Heatmap showing differentially expressed genes (DESeq2, *n* = 265, *P*_adj_ ≤ 0.05) between individuals (*n* = 14) from humic and clear-water lakes. Yellow and violet colour correspond to an increased and decreased transcript abundance, respectively, in the clear-water lakes. The bar-plot above the figure illustrates dissolved organic carbon (DOC) concentration (mg/l) in each studied lake. The table indicates the number of reads that were assigned to the order Strigeidida and the superfamily Diplostomoidea; the results of PCR amplification of diplostomid-specific *cox*1 gene in humic and clear-water lakes; and the proportion of diplostomid-specific *cox*1 reads assigned to the genus *Diplostomum* and *Tylodelphys* and to the species *Tylodelphys clavata* in four clear-water lakes

Next, we rigorously filtered the sequences to further minimize technical artefacts that could lead to overestimation of haplotype diversity [25, 54]. As the majority of parasite sequences belonged to *Tylodelphys clavata* (mean = 83.6%; median = 94.0%; Fig. 1; Additional file 2: Table S2), we further characterized the intraspecific variation of this species. All of the sequences assigned to *T. clavata* were extracted and clustered with cd-hit 4.7 [55, 56] using 100% similarity to remove redundancy and exclude unique sequences, as the latter could appear due to technical PCR or sequencing errors. For the subsequent analyses, we used only representative sequences of the clusters with more than 2.5% of the total number of sequences assigned to *T. clavata* per sample. In addition, the haplotypes that were observed only in a single sample were excluded, as they may represent sequencing artefacts [25, 54]. However, this procedure might potentially eliminate rare haplotypes from the subsequent analysis. The final dataset contained 348 *T. clavata* sequences from 113 eye samples (79 individuals). For comparative purposes we added 7 partial sequences of *T. clavata cox*1 retrieved from GenBank (accession numbers: KR271473.1; KR271475.1; KR271480.1; KT751175.1; KT768015.1; KT961707.1; and KY271544.1). All sequences were aligned using Muscle 3.8.31 [57], and the NCBI sequences were trimmed to the same size of the *cox*1 fragments generated during NGS sequencing, using BioEdit 7.2.5 [58]. To visualize the relationships among haplotypes, a TCS haplotype network [59] was generated using PopART1.7 (http://popart.otago.ac.nz).

## Results

### Initial insights from eye transcriptomes

Altogether, 94% of the reads from the 14 RNA-seq libraries generated from whole-eye tissue were mapped to the reference perch genome (Additional file 2: Table S3). In total, 265 perch genes were found to be differentially expressed (*P*_adj_ ≤ 0.05) between fish originating from humic and clear-water lakes (Fig. 1, Additional file 2: Table S4). Gene Ontology (GO) analysis indicated that the differentially expressed genes were enriched for 69 GO process terms (GOrilla, FDR ≤ 0.05), with the top 3 terms (FDR < 1.14*10^−5^), consisting of immune system process (GO:0002376, *n* = 50), adaptive immune response process (GO:0002250, *n* = 15) and immune response process (GO:0006955, *n* = 27, Additional file 2: Table S5).

Evaluation of unmapped whole-eye RNA-seq reads revealed that the samples from 4 out of 6 clear-water lakes contained sequences that originated from parasitic flatworms of the order Strigeidida or/and superfamily Diplostomoidea, but no reads were detected in any of the 8 humic water lakes (Fig. 1).

### PCR-based validation

The ~500-bp Diplostomidae *cox*1 gene fragment was successfully amplified in 95 of the 212 individuals additionally sampled in 2017 (142 of 384 eye samples; Additional file 2: Table S1). All of the collected samples from the 8 humic lakes were free of diplostomid parasites, whereas perch from 4 of the 5 clear-water lakes were infected. Infection prevalence was very high in 3 clear-water lakes (prevalence 96–100%, Fig. 1, Additional file 2: Table S1).

### Targeted metabarcoding of eye parasites

The majority of the PCR-positive eye samples produced a large number of sequences of parasites belonging to the Diplostomidae (mean number of reads = 53,171; median = 39,651). In total, 99.3% of the sequences were assigned to the superfamily Diplostomoidea. The majority of sequences (mean = 83.6%) were assigned to *Tylodelphys clavata* (Additional file 2: Table S2) while a small number of sequences were assigned to 3 species from the genus *Diplostomum* (*D. baeri* complex sp. 2 SAL-2014 (*n*_eyes_ = 7), *D. spathaceum* (*n*_eyes_ = 4) and *D. pseudospathaceum* (*n*_eyes_ = 7; Additional file 2: Table S2).

**Fig. 2:**
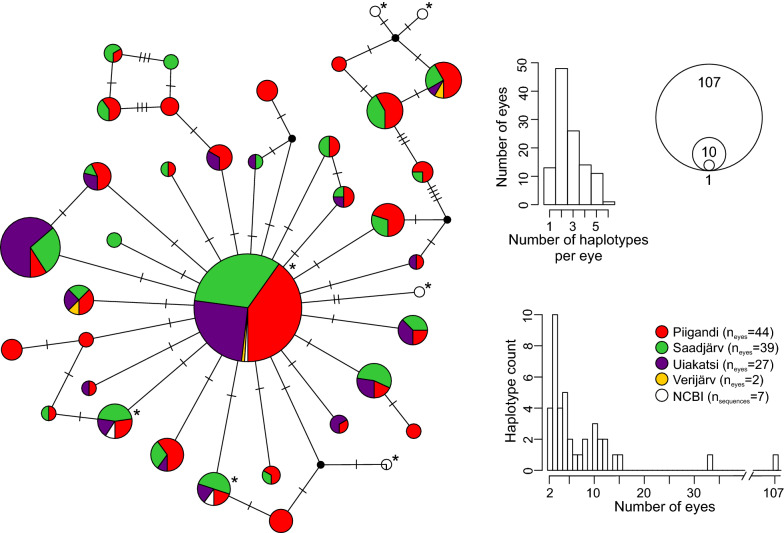
Haplotype network of *cox*1 sequences for *Tylodelphys clavata*. Circle size is proportional to the frequency of each haplotype. Perch populations from different lakes are represented by different colours. The haplotypes that include NCBI sequences are highlighted with a star; *n*_eyes_ refers to the number of eye samples; *n*_sequences_ refers to the number of sequences from the NCBI database. The insert histograms illustrate the number of haplotypes observed per eye and the frequency of haplotypes

Altogether, 34 distinct *T. clavata* haplotypes were identified in the 113 analysed eye samples collected from 79 perch; of these, 4 haplotypes were identical to published GenBank sequences. The most common haplotype was found in 107 samples, while the other haplotypes were observed in 2 to 16 samples (Fig. 2). The majority of the eyes contained 1 to 5 haplotypes. Most of the haplotypes formed a genetically close star-like network, whereas two smaller haplotype groups were more distant from the former (Fig. 2). There was no evidence of strong genetic structuring, as common haplotypes were present in all 4 lakes.

## Discussion

The extent to which extrinsic environmental conditions shape host-pathogen coevolution and contribute to the emergence of locally adapted populations are currently poorly understood. Here, we demonstrate how integrated use of complementary NGS approaches can provide novel insights on such complex associations [2, 15, 18, 60]. By analysing both host-specific and unmapped whole-eye RNA-seq reads, we discovered that perch individuals from humic and clear-water lakes differ in immune system related gene expression, and that this difference could be explained by contrasting diplostomid parasite pressure between the two habitats. We subsequently developed a targeted metabarcoding approach to further investigate the molecular diversity of this parasite group. We found that *T. clavata* is the dominant eye parasite in perch, with high prevalence and haplotype diversity in the four clear-water lakes. While high prevalence and abundance of *T. clavata* in perch has been observed earlier [26], our work provides support for the hypothesis that the humic environment is unfavourable at least for this diplostomid eye parasite species to successfully complete its life-cycle. Moreover, to the best of our knowledge, we show for the first time that in addition to the head-kidney, which is the main lymphoid organ involved in piscine immune defence [61], the presence of eye parasites also alters the expression patterns of a number of host immune genes measured from the whole eye.

### Differential expression of immune genes

The adaptive importance of gene functions can be studied by analysing gene expression differences in an ecological context [62]. Among the genes that were differentially expressed between eyes of perch from clear-water and humic lakes, those with immune system-related functions were strongly overrepresented. Differentially expressed genes included interferons, interleukins, and other proteins (e.g. interferon regulatory factor 1, interferon induced proteins, interleukin-8 like protein, MHC class II beta subunit and T cell antigens) that are involved in immune cell activation and antigen presentation.

In wild populations, immune system genes are often found to be at the very centre of evolutionary change [63–65]. Nevertheless, the expression of immune-related genes in the perch eye was initially unexpected, as traditionally the eye has been thought to be an “immunoprivileged” organ [66–69]. However, accumulating evidence has started to paint a more complex picture of ocular immunity by, for instance, showing that leucocytes can selectively penetrate the retina-blood barrier [70], and that immune system related genes are expressed in various eye microhabitats [71, 72].

One interesting differentially expressed gene found in our study is catalase (CAT; EC1.11.1.6), which is a principal enzyme in antioxidant pathway that functions by converting reactive H_2_O_2_ to H_2_O and O_2_. CAT showed a marked downregulation in clear-water lakes (Additional file 2: Table S5). CAT enzymatic activity has been studied in various compartments of the eye in humans and model organisms [73, 74], and reduced CAT activity was linked to decreased parasitosis [75]. However, because here we have analysed gene expression of the whole eye rather than that of specific eye structures and tissues, and without blood expression data for contrast, we cannot determine the extent to which the observed expression differences are driven by the processes in blood *versus* internal eye structures. Nevertheless, our results indicate that *T. clavata* is most likely influencing immune gene expression patterns of the host. Most of the current (and limited) information we have on eye immunity comes from mammalian models; we know very little about immune processes in the eye of other taxa [68, 69, 76, 77]. More studies targeting multiple eye tissues [78] are therefore clearly needed to evaluate the “immunopriviliged” status of fish eyes in response to eye parasites.

### Humic lakes as eye parasite-free environment for perch

To explain the excess of differentially expressed immune-related genes between humic and clear-water perch populations, we hypothesized that observed differences in transcript abundances may be driven by eye parasites. In order to test the potential link between humic substances and occurrence of diplostomid parasites, we scanned the proportion of RNA-seq reads that were not mapped to the perch genome. For individuals originating from humic lakes, none of the unmapped RNA-seq reads were assigned to the Diplostomoidea. This initial result was later confirmed with PCR-based screening of additional samples collected the following year when a very high prevalence of diplostomid parasites was observed in four out of six clear-water lakes. This result is consistent with previous studies in perch and other fish species, which showed the absence of some parasite taxa in potentially challenging habitats [34, 79, 80]. Diplostomid parasites have a complex life-cycle with three hosts and free-living stages, making this group particularly sensitive to biotic and abiotic elements of their environment. Because both clear-water and humic lake pairs are in very close geographical proximity (see Additional file 1: Figure S1), the difference in parasite prevalence cannot be explained by the lack of dispersal opportunities for the parasite [81]. The most obvious difference between lakes is their colour, which is tightly linked to water chemistry, particularly DOC and pH (Pearsons’s *r* = − 0.64, *P* = 0.003). Monitoring data of gastropod diversity indicated their absence in most of the studied humic lakes. In clear-water lakes, however, at least one species of gastropod was recorded (*Lymnaea* spp. or *Radix* spp.), which are both considered as first intermediate hosts for diplostomid species. Moreover, high density of underwater vegetation in clear-water lakes likely supports high density of gastropods, while humic lakes are typically very poor in aquatic vegetation. Taken together, this suggests that interactive effects driven by the humic content on diplostomid parasite free-living stage and the lack of the first intermediate gastropod host [34, 82] most likely create a ‘life-cycle bottleneck’ for the parasite [81].

### Cryptic diversity in *T. clavata*

DNA analysis of naturally pooled fish-eye parasites has previously been used in combination with pyrosequencing [29]. However, the early attempts to harness the power of NGS for intra- and interspecific analysis were severely hampered by very short read length (e.g. only 22 bp were sequenced in [29]). In the present study, we developed targeted metabarcoding of a longer (~ 500 bp) diplostomid-specific *cox*1 fragment for whole-eye parasite community analysis. Using a conservative approach of eliminating singletons and rare reads we assigned most of the *cox*1 fragments to *T. clavata*.

We observed high *T. clavata* haplotype diversity among the studied lakes, as well as a lack of genetic structuring, consistent with previous studies [32, 83]. Together, this suggests that *T. clavata* forms a large well-connected population system, as is expected for parasites with highly mobile definitive hosts such as piscivorous birds [83]. The high haplotype diversity in *T. clavata* observed here also suggests that earlier sequencing efforts have likely managed to capture only a fraction of the intraspecific genetic diversity. It is likely that this finding also holds for other diplostomid species; current molecular studies of fish eye flukes are typically based on analysis of less than a hundred individually sampled parasites (but see [32]), yet a single fish eye may harbour hundreds of parasites (e.g. [26]). Thus, it was not surprising that the developed diplostomid metabarcoding approach revealed, for the first time, an extensive intraspecific diversity in *T. clavata.* Our study also showed that the majority of perch were infected by several *T. clavata* haplotypes. The latter result would indicate continual infection by different haplotypes that co-exist in the same lakes—a result also observed for liver flukes [84, 85].

## Conclusions

Taken together, this study demonstrates how components of the abiotic environment drastically shape common parasite communities and host immune response, highlighting the significance of analysing results of host-parasite studies in an ecological context. In addition, our study illustrates the utility of integrating RNA-seq and targeted metabarcoding approaches in host-parasite community studies. The high intraspecific diversity of *T. clavata* recovered from our targeted metabarcoding approach suggests that NGS of naturally pooled samples represents an efficient and powerful strategy for shedding light on cryptic diversity of eye parasites.

## Supplementary information

**Additional file 1: Figure S1.** A map illustrating the geographical location of sampled lakes. The numbers correspond to: 1, Holvandi Kivijärv; 2, Virosi; 3, Partsi Saarjärv; 4, Heisri Mustjärv; 5, Kuulma; 6, Loosalu; 7, Matsimäe Pühajärv; 8, Meelva; 9, Paidra; 10, Hino; 11, Verijärv; 12, Saadjärv; 13, Uiakatsi; and 14, Piigandi. Humic and clear-water lakes are shown as filled black and white circles, respectively.

**Additional file 2: Table S1.** Sampling location, year, number of samples for RNA-seq NGS, number of samples for parasite screening. **Table S2.** Samples, location, Illumina reads statistics and the main parasite OTUs as revealed by Kraken and RDP. **Table S3.** RNA-seq mapping statistics. **Table S4.** List of 265 differentially expressed genes in perch identified by RNA-seq (*P*_adj_ < 0.05), sorted by log2 fold change. **Table S5.** List of gene ontology (GO) terms with significant (FDR ≤ 0.05) enrichment among the differentially expressed genes. **Table S6.** Absorbance ratios, specific ultraviolet absorbance and specific colour absorbance for studied lakes.

**Additional file 3: Text S1.** Expanded methods description.

## Data Availability

Transcriptome reads of *P. fluviatilis* eye are available in the NCBI SRA (SRR10441590-SRR10441602 and SRR7091762) as a part of BioProjects PRJNA589499 and PRJNA450919, respectively. Short Illumina linked-reads of Diplostomidae mtDNA cytochrome *c* oxidase subunit 1 fragment are available in the NCBI SRA (SRR10490070-SRR10490211) as a part of BioProject PRJNA590324.

## Abbreviations

CAT: catalase
cox1: cytochrome *c* oxidase subunit 1
DOC: dissolved organic carbon
GO: Gene Ontology
NGS: next generation sequencing
