# Supplementary figures and images for: Humic-acid-driven escape from eye parasites revealed by RNA-seq and target-specific metabarcoding
Source: Parasit Vectors. 2020 Aug 28;13:433. doi: 10.1186/s13071-020-04306-9 (PMC7456052; doi:10.1186/s13071-020-04306-9)

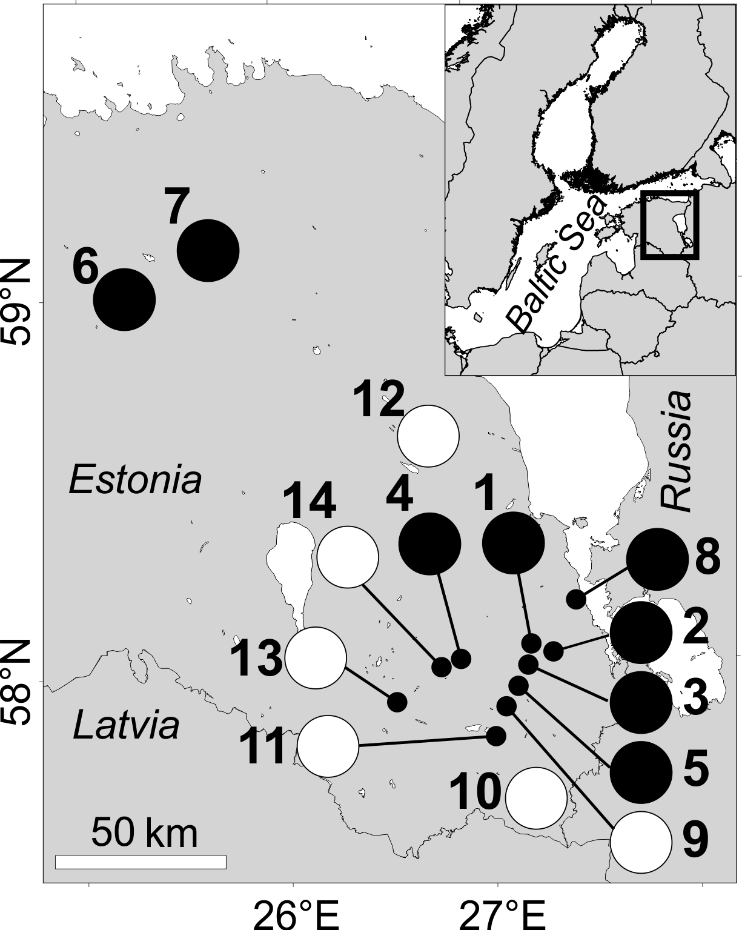

Supplement: Supplementary file 1 — Additional file 1: Figure S1. A map illustrating the geographical location of sampled lakes. The numbers correspond to: 1, Holvandi Kivijärv; 2, Virosi; 3, Partsi Saarjärv; 4, Heisri Mustjärv; 5, Kuulma; 6, Loosalu; 7, Matsimäe Pühajärv; 8, Meelva; 9, Paidra; 10, Hino; 11, Verijärv; 12, Saadjärv; 13, Uiakatsi; and 14, Piigandi. Humic and clear-water lakes are shown as filled black and white circles, respectively. [file 13071_2020_4306_MOESM1_ESM.tif]
