# Supplementary material for: Humic-acid-driven escape from eye parasites revealed by RNA-seq and target-specific metabarcoding
Source: Parasit Vectors. 2020 Aug 28;13:433. doi: 10.1186/s13071-020-04306-9 (PMC7456052; doi:10.1186/s13071-020-04306-9)
Supplement: Supplementary file 3 — Additional file 3: Text S1. Expanded methods description. [file 13071_2020_4306_MOESM3_ESM.docx]

**Additional file 3**

**Humic-acid-driven escape from eye parasites revealed by RNA-seq and target-specific metabarcoding**

**Methods**

**Sampling of perch**

The Eurasian perch (*Perca fluviatilis*) from eight humic and six clear-water lakes in Estonia (also see Additional file 1: Figure S1 and Additional file 2: Table S1) were caught using gill- and seine nets in September of 2016. Nets were constantly monitored and live fish were immediately euthanized by an overdose of tricaine methanesulfonate (MS-222) before sampling. A whole left eye was dissected from the individual perch using a sterile scalpel and instantly stored in liquid nitrogen. The additional sampling of perch from the same lakes for PCR validation and NGS was performed in 2017 using the same approach (Additional file 2: Table S1). Information about gastropods diversity (presence/absence) was obtained from Estonian Environmental Monitoring database (https://www.keskkonnaagentuur.ee). Gastropods taxa were identified to the species level using morphological criteria by a specialist.

**Water samples**

Surface water samples were collected simultaneously with fish sampling in September of 2016. The pH and dissolved organic carbon (DOC) concentrations (mg/l) were determined for all the lakes (Table 1). In addition, absorbance and fluorescence of the filtered water samples were measured to obtain nine different spectral parameters: A_250_/A_365_, A_254_/A_436_, A_365_/A_465_ and A_465_/A_665_, SUVA_254_, SUVA_285_ and SCOA_436_ (L mg^−1^ m^−1^), and fluorescence index and freshness index (Additional file 2: Table 6). DOC and applied spectral parameters were used to characterize the humic content of the studied lakes. All analyses were performed as described in [1] using the same equipment and laboratory facilities.

**RNA extraction and library preparation**

Total RNA was extracted from the frozen homogenized eyeball (30 mg of tissue) of one female perch per lake sampled in 2016, using NucleoSpin® RNA extraction kit (MACHEREY-NAGEL, Duren, Germany). The quality of the total RNA sample was evaluated using Bioanalyzer 2100 (Agilent) electrophoresis and sample concentration was measured with Nanodrop ND-2000 (Thermo Scientific™). The library was prepared from 300 ng of total RNA according to Illumina TruSeq® Stranded mRNA Sample Preparation Guide (part no. 15031047) to generate a 300 bp insert size library. The library was sequenced using Illumina HiSeq 3000 (2 x 75 bp configuration, half a lane) in the Finnish Functional Genomics Centre (Turku, Finland).

**Read quality**

Sequencing data were sorted by individual and trimmed removing indexing adaptors at the sequencing facility. Reads quality was examined using FastQC ver. 0.11.8 [2] and quality trimming was performed using Trimmomatic ver.0.35 [3] with the following parameters: ILLUMINACLIP:/trimmomatic/adapters/TruSeq3-PE.fa:2:30:10 SLIDINGWINDOW:5:25 MINLEN:50. Reads less than 50 bp length and the average quality below 25 within a window of five nucleotides were excluded from the dataset.

**Read mapping**

The reads that passed the quality control were mapped on the reference genome of *Perca fluviatilis* ([4]; GCA_003412525.1) using hisat2 ver. 2.1.0 [5] with default parameters. The sam files were processed with samtools ver.1.4 [6]. The only mapped (primary aligned) reads were extracted to bam-files for subsequent differential expression analyses (-F 260).

**Differential expression analysis for sequence count data**

The ranges for genomic features of different types were generated from the gff file containing perch reference genome annotation records using the function makeTxDbFromGFF from the GenomicFeatures package ver.3.8 [7] in R 3.3.4 [8]. Further, the overlapping of reads with annotation ranges of interest were counted for each sample using the summarizeOverlaps function of the GenomicAlignments package ver.3.8 [7]. The read counting was performed for exonic gene regions in a non-strand-specific manner while ignoring overlaps among different genes and the results were stored in the raw read count table. Differential expression analysis was performed using the DEseq2 package ver.1.22.2 [9]. Fold changes (FC) in the abundance of perch transcripts between two groups of lakes (humic vs. clear-water) were determined. All genes with an adjusted p-value ≤ 0.05 [10] were considered as significantly differentially expressed between two types of lakes.

**Gene Ontology (GO) analysis**

Human orthologue gene symbols were searched using complete gene names in NCBI. GO-enrichment analysis of differentially expressed genes against all orthologue genes symbols as a background was performed using Gorilla ([11]; http://cbl-gorilla.cs.technion.ac.il/). The GO terms with FDR ≤ 0.05 were considered as significant.

**Examining species composition of the unmapped reads**

As the GO term analysis revealed large number of DE genes related to immune function (see Results section and Additional file 2: Table S5) we subsequently examined if the observed pattern could be associated with the occurrence of eye parasites in clear-water lakes. First, we extracted unmapped reads from each eye sample to further determine their origin (samtoolsfasta -@ 12 -f 4 aligned.bam > unmapped.fasta). The redundancy among the unmapped reads was reduced using cd-hit ver. 4.7 [12, 13]. When two or more reads showed 99% or higher similarity all but the longest read were removed to generate a non-redundant set of unmapped reads (cd-hit-est -c 0.99). NCBI’s blastn ver. 2.6.0 [14] was further applied to align the non-redundant sets of unmapped reads to the sequences in a non-redundant nucleotide database (ftp://ftp.ncbi.nlm.nih.gov/blast/db/FASTA/nr.gz; downloaded December 2018). Default parameters were used, except maximum number of targets to report and the number of high-scoring segment pairs (HSPs) reported per hit were set to five (-max_target_seqs 5 -max_hsps 5) and e-value was set to 0.001 (-evalue 0.001). To reveal the presence of the eye fluke parasites’ sequences (of the Digenea class of Trematoda and of the *Diplostomidae* family) among the unmapped reads the taxonomic analysis of blastn outputs was processed in Megan Community Edition ver. 6.8.18 [15].

**Amplification and electrophoresis of mitochondrial cytochrome c oxidase I (*cox1*) in the family Diplostomidae**

As the species composition analysis of the unmapped RNA-reads showed the presence of the reads assigned to Strigeidida order or/and Diplostomoidea superfamily only in the clear-water lakes, we further screened additional eye samples from 212 perch (Additional file 2: Table S1). We used modified diplostomid-specific primers for *cox1* [16] that included linkers for Illumina-compatible adapters at their 5′ ends ([17, 18]; Plat-diploCOX1_ILL_F: TCGTCGGCAGCGTCAGATGTGTATAAGAGACAGCGTTTRAATTATACGGATCC; Plat-diploCOX1_ILL_R: GTCTCGTGGGCTCGGAGATGTGTATAAGAGACAGAGCATAGTAATMGCAGCAGC; *cox1*-specific sequences are underlined). The DNA was extracted from the whole perch eye according to [19]. The concentration of DNA extracts was measured using Nanodrop ND‐1000 (Thermo Scientific™). PCR was performed in 10 µl total volume and included 2×QMP-reagent (QIAGEN Multiplex PCR Kit), 3.3 pmol of each primer and approximately 100-200 ng of DNA template. The PCR cycling conditions included 15 min in 95°C, then 30 cycles of 30 s in 95°C, 30 s in 58°C and 30 s in 72°C, ending with a final extension of 3 min at72°C. The electrophoresis of PCR products was performed in 1.5% agarose gels for 30 min (40-50 V) and gels were visualized using Chemidoc XRS (BioRad) to detect ca. 500bp diplostomid-specific *cox1* amplicon. For 172 individuals both eyes were screened, while for the remaining 40 individuals, only left eye was examined.

**Next generation sequencing of *cox1* in Diplostomidae community within perch eye**

To further determine the specific parasite species, 142 PCR-positive samples were chosen for sequencing (Additional file 2: Table S1 and Table S2). For library preparation PCR-products from the reaction described above were purified using SPRI beads and used for the second PCR to attach Illumina adapters and unique individual indexes following the modified PCR protocol described in [18]: for a reaction volume of 12.5 μl, 6.25 μl KAPA HiFi HotStart MasterMix (product nr KK2602, KAPA Biosystems, Wilmington, Massachusetts, USA), 3.75 pmol of each primer, 1.25 μl of sterile deionized water and 2 μl of purified locus‐specific PCR product were mixed. The PCR cycling conditions were similar to those described in [18] except that the extension step was 20 s. After the indexing PCR, 2 μl of each indexed sample were pooled together and purified using SPRI beads. The quality of library pool was ensured using Agilent Bioanalyzer 2100 and the library pool concentration was measured with Qubit DNA HS kit. The samples were sequenced in a single run with Illumina MiSeq instrument (Illumina Inc., San Diego, California, USA) by the Turku Centre for Biotechnology, Turku, Finland, using a v3 sequencing chemistry. Paired-end sequencing with 2 x 300 bp read length was used with 8 bp dual index run.

**Processing of the *cox1* reads**

The demultiplexed paired-end reads were merged using PEAR ver. 0.9.6 [20] with default parameters, except that the assembly range was set between 400 and 600 bp. The merged reads containing non-complete primer tails (~ 12%) were excluded from the following analyses (Additional file 2: Table S2). The both forward and reverse primer tails were removed from the sequences using cutadapt ver. 1.10 [21] with default settings. For robust downstream analysis only the samples containing ≥1000 filtered and merged sequences [22] were retained (115 of 142; Additional file 2: Table S2).

Taxonomic classification of parasite sequences was performed with Kraken ver. 2.0.6-beta [23], using a custom database generated from the available *cox1* gene sequences of the phylum Plathyhelmintes. The sequences were extracted from the NCBI’s non-redundant nucleotide database (ftp://ftp.ncbi.nlm.nih.gov/blast/db/FASTA/nr.gz; downloaded December 2018) and converted to a Kraken database using kraken2-build. In addition, to validate the Kaken results with probabilistic approach, the parasite sequences were classified by applying a naïve Bayesian classifier using RPD ver. 11.5 [24] as described in [25]. The RDP database was generated from the same Plathyhelmintes NCBI sequences after adding full taxonomy path in Geneious Prime ver. 2019.2 [26]. A 95% probability cut-off was applied for RDP results to assign parasite sequences to a genus/species.

As both taxonomic classifiers showed consistent results, the further analyses was based only on Kraken classification. To avoid biases related to unequal number of reads per sample [27], the evidence of the presence of a particular parasite genus/species in a sample was considered highly supported if ≥ 5% of the sequences were assigned to a parasite genus/species per eye sample. Inevitably, this threshold represents a trade-off between maximizing inclusion of real species and excluding low-level background noise. Here, we chose a conservative threshold to avoid incorrect assignments but this may, in theory, exclude rare taxa.

As the analyses of Kraken results (Fig.1; Additional file 2: Table S2) revealed that the most of parasite sequences came from *Tylodelphys clavata* (mean=82.7%; median=93.9%), the intraspecific diversity of this parasite species was investigated further. First, for each eye sample we extracted all the sequences assigned to *T. clavata*. All the sequences containing unknown bases were excluded. Next, sequences were clustered with cd-hit ver. 4.7 [12, 13] using 100% similarity (-c 1) to remove redundancy and exclude unique sequences, as the latter could appear due to technical PCR or sequencing errors. For the subsequent analyses we used only representative sequences of the cd-hit clusters with more than 2.5% of the total number of sequences (excluding singletons) per sample assigned to *T. clavata*. We also excluded haplotypes that here observed only in a single sample, as these may represent sequencing artefacts. On the other hand, this procedure potentially eliminated rare haplotypes from subsequent analysis. The final dataset contained 348 *T. clavata* sequences from 113 eye samples. For comparative purpose we added 7 partial sequences of *T. clavata* *cox1* gene deposited in GenBank (accession numbers: KR271473.1, KR271475.1, KR271480.1, KT751175.1, KT768015.1, KT961707.1, KY271544.1). All the sequences were aligned using Muscle ver. 3.8.31 [28] and NCBI sequences were trimmed using BioEdit ver. 7.2.5 [29] to correspond the size of the *cox1* fragments from MiSeq (462 bp). To visualize the relationships among haplotypes the TCS [30] haplotype network was generated using PopART ver. 1.7 (http://popart.otago.ac.nz).

**References**

1. Sepp M, Kõiv T, Nõges P, Nõges T. The role of catchment soils and land cover on dissolved organic matter (DOM) properties in temperate lakes. *J Hydrol.* 2019;570:281–91.doi:10.1016/j.jhydrol.2019.01.012

2. Andrews S. *FastQC: a quality control tool for high throughput sequence data*. 2018. https://www.bioinformatics.babraham.ac.uk/projects/fastqc/.

3. Bolger AM, Lohse M, Usadel B. Trimmomatic: a flexible trimmer for Illumina sequence data. *Bioinformatics.* 2014;30:2114–20. (doi:10.1093/bioinformatics/btu170)

4. Ozerov MY, Ahmad F, Gross R, Pukk L, Kahar S, Kisand V, et al. Highly continuous genome assembly of Eurasian perch (*Perca fluviatilis*) using linked-read sequencing. *G3 Genes Genomes Genet.* 2018;8: 3737–43.doi:10.1534/g3.118.200768

5. Kim D, Langmead B, Salzberg SL. HISAT: a fast spliced aligner with low memory requirements. *Nat Methods.* 2015;12:357–60.doi:10.1038/nmeth.3317

6. Li H, Handsaker B, Wysoker A, Fennell T, Ruan J, Homer N, *et al.* The sequence alignment/map format and SAMtools. *Bioinforma Oxf Engl.* 2009;25:2078–79. doi:10.1093/bioinformatics/btp352

7. Lawrence M, Huber W, Pagès H, Aboyoun P, Carlson M, Gentleman R, et al. Software for computing and annotating genomic ranges. *PLoS Comput Biol.* 2013;9: e1003118. doi:10.1371/journal.pcbi.1003118

8. R Core Team. *R: A Language and Environment for Statistical Computing.* Vienna: R Foundation for Statistical Computing. 2018.

9. Love MI, Huber W, Anders S. Moderated estimation of fold change and dispersion for RNA-seq data with DESeq2. *Genome Biol.* 2014;15:550. doi:10.1186/s13059-014-0550-8

10. Benjamini Y, Hochberg Y. Controlling the false discovery rate: a practical and powerful approach to multiple testing. 1995; doi:10.1111/j.2517-6161.1995.tb02031.x

11. Eden E, Navon R, Steinfeld I, Lipson D, Yakhini Z. GOrilla: a tool for discovery and visualization of enriched GO terms in ranked gene lists. *BMC Bioinformatics.* 2009;10:48. doi:10.1186/1471-2105-10-48

12. Fu L, Niu B, Zhu Z, Wu S, Li W. CD-HIT: accelerated for clustering the next-generation sequencing data. *Bioinforma Oxf Engl.* 2012;28:3150–52. doi:10.1093/bioinformatics/bts565

13. Li W, Godzik A. Cd-hit: a fast program for clustering and comparing large sets of protein or nucleotide sequences. *Bioinforma Oxf Engl.* 2006;22:1658–59. doi:10.1093/bioinformatics/btl158

14. Boratyn GM, Camacho C, Cooper PS, Colouris G, Fong A, Ma N, et al. BLAST: a more efficient report with usability improvements. *Nucleic Acids Res.* 2013;41:W29-33. doi:10.1093/nar/gkt282

15. Huson DH, Beier S, Flade I, Górska A, El-Hadidi M, Mitra S, et al. MEGAN community edition - interactive exploration and analysis of large-scale microbiome sequencing data. *PLoS Comput Biol.* 2016;12:e1004957. doi:10.1371/journal.pcbi.1004957

16. Moszczynska A, Locke SA, McLaughlin JD, Marcogliese DJ, Crease TJ. Development of primers for the mitochondrial cytochrome c oxidase I gene in digenetic trematodes (Platyhelminthes) illustrates the challenge of barcoding parasitic helminths. *Mol Ecol Resour.* 2009:9:s1:75–82. doi:10.1111/j.1755-0998.2009.02634.x

17. Clarke LJ, Czechowski P, Soubrier J, Stevens MI, Cooper A. Modular tagging of amplicons using a single PCR for high-throughput sequencing. *Mol Ecol Resour.* 2014;14:117–121. doi:10.1111/1755-0998.12162

18. Kaunisto KM, Roslin T, Sääksjärvi IE, Vesterinen EJ. Pellets of proof: First glimpse of the dietary composition of adult odonates as revealed by metabarcoding of feces. *Ecol Evol.* 2017;7:8588–98. doi:10.1002/ece3.3404

19. Aljanabi SM, Martinez I. Universal and rapid salt-extraction of high quality genomic DNA for PCR-based techniques. *Nucleic Acids Res.* 1997;25:4692–93.

20. Zhang J, Kobert K, Flouri T, Stamatakis A. PEAR: a fast and accurate Illumina Paired-End reAdmergeR. *Bioinforma Oxf Engl.* 2014;30;614–20. doi:10.1093/bioinformatics/btt593

21. Martin M. Cutadapt removes adapter sequences from high-throughput sequencing reads. *EMBnet journal.* 2011;17:10–12. doi:10.14806/ej.17.1.200

22. Koskinen J, Roslin T, Nyman T, Abrego N, Michell C, Vesterinen EJ. Finding flies in the mushroom soup: Host specificity of fungus-associated communities revisited with a novel molecular method. *Mol Ecol.* 2019;28:190–202. doi:10.1111/mec.14810

23. Wood DE, Salzberg SL. Kraken: ultrafast metagenomic sequence classification using exact alignments. *Genome Biol.* 2014;15:R46. doi:10.1186/gb-2014-15-3-r46

24. Wang Q, Garrity GM, Tiedje JM, Cole JR. Naive Bayesian classifier for rapid assignment of rRNA sequences into the new bacterial taxonomy. *Appl Environ Microbiol.* 2007;73:5261–67. doi:10.1128/AEM.00062-07

25. Porter TM, Hajibabaei M. Automated high throughput animal CO1 metabarcode classification. *Sci Rep.* 2018;8:1–10. doi:10.1038/s41598-018-22505-4

26. Kearse M, Moir R, Wilson A, Stones-Havas S, Cheung M, Sturrock S, et al. Geneious Basic: an integrated and extendable desktop software platform for the organization and analysis of sequence data. *Bioinforma Oxf Engl.* 2012;28:1647–49. doi:10.1093/bioinformatics/bts199

27. Deagle BE, Thomas AC, McInnes JC, Clarke LJ, Vesterinen EJ, Clare EL et al. Counting with DNA in metabarcoding studies: How should we convert sequence reads to dietary data? *Mol Ecol.* 2019;28:391–406. doi:10.1111/mec.14734

28. Edgar RC. MUSCLE: multiple sequence alignment with high accuracy and high throughput. *Nucleic Acids Res.* 2004;32:1792–97. doi:10.1093/nar/gkh340

29. Hall A. BioEdit: a user-friendly biological sequence alignment editor and analysis program of Windows 95/98/NT. 1999. doi:10.1021/bk-1999-0734.ch008

30. Templeton AR, Crandall KA, Sing CF. A cladistic analysis of phenotypic associations with haplotypes inferred from restriction endonuclease mapping and DNA sequence data. III. Cladogram estimation. *Genetics.* 1992;132:619–33.
